# Supporting Success: Qualitative Study of Mentoring CASC Candidates Through Structured Exam Preparation

**DOI:** 10.1192/bjo.2025.10285

**Published:** 2025-06-20

**Authors:** Debora Macedo, Isabel Mark

**Affiliations:** South West London and St George’s Mental Health NHS Trust, London, United Kingdom

## Abstract

**Aims:** Differential attainment is recognised as a key factor in MRCPsych examinations, with gender, training status, ethnicity and international medical graduate (IMG) status significantly influencing CASC outcomes. Anxiety and other unique challenges faced by IMGs can contribute to performance gaps. Educational theory emphasises multimodal frameworks and peer-led mentoring can enhance learning outcomes and alleviate exam-related anxiety, thereby narrowing these disparities. This study evaluates the impact of a structured, peer-led mentoring approach on CASC success, aiming to target the unmet needs of IMG candidates and develop effective, scalable strategies for reducing performance disparities.

**Methods:** In 2024, a structured mentoring programme was designed at South West London and St George’s Mental Health NHS Trust, supporting CASC candidates preparing for their first attempt. The cohort included 8 trainees of both genders, from different ethnic and training backgrounds. The mentoring framework integrated the following components:

Initial planning with clear expectations and timeline.

Practice framework of all station types and a consistent practice schedule, which increased in frequency and intensity closer to the exam.

Station-specific flowcharts and time-saving strategies provided to enhance efficiency.

Ongoing support with practical advice on anxiety management and exam logistics.

Full mock examinations conducted with detailed feedback.

**Results:** All participants passed the CASC on their first attempt. Qualitative feedback, analysed using Braun and Clarke’s six-stage thematic framework, revealed the following key themes:

Clarity and Structure: Participants highlighted that a structured timetable and clear objectives improved focus and confidence, making the workload more manageable and progress tangible.

Practical Resources: Flowcharts and checklists were deemed highly effective in simplifying complex tasks with multiple potential approaches.

Emotional Support: Regular peer interactions reduced anxiety and fostered a collaborative learning environment.

Feedback and Reflection: Mock exams with detailed feedback were viewed as instrumental in identifying strengths and areas for improvement.

**Conclusion:** This study demonstrates the potential for peer-led mentorship programmes during CASC examination preparation, minimising candidate anxiety and helping to foster equitable outcomes. Results highlighted the benefits of structure, consistency, targeted support and personalised feedback, tailored to the diverse needs of the trainees. Further research with larger cohorts is recommended to quantitatively evaluate the generalisability of these findings. An expanded version of this mentoring programme might be introduced within the Trust. Establishing such a programme could not only improve exam pass rates but also foster a more inclusive and supportive training environment within psychiatry.

